# Predictors of tuberculosis treatment outcomes among people living with HIV in some States in Nigeria

**DOI:** 10.11604/pamj.2024.47.149.35719

**Published:** 2024-03-28

**Authors:** Olalere Samuel Olajide, Prosper Okonkwo, Oluseye Ajayi, Dickson Adetoye, Olabanjo Okunlola Ogunsola, Olorunfemi Ogundele, Oluwasogo Elujide, Folake Adurogbola, Plang Jwanle

**Affiliations:** 1 APIN Public Health Initiatives, Ibadan, Nigeria,; 2 APIN Public Health Initiatives, Abuja Federal Capital Territory, Nigeria,; 3 University of Medical Sciences Ondo, Ondo State, Nigeria

**Keywords:** Tuberculosis, TB/HIV co-infection, isoniazid, antiretroviral therapy, treatment outcome

## Abstract

**Introduction:**

tuberculosis (TB) and Human Immunodeficiency Virus (HIV) remain major public health threats globally and worse when they co-exist in susceptible individuals. The study examined TB treatment outcomes and their predictive factors among people living with HIV (PLHIVs).

**Methods:**

a review of TB/HIV co-infected patients who had TB treatments across comprehensive antiretroviral therapy (ART) sites with ≥500 patients was conducted in seven United States of America President's Emergency Plan for AIDS Relief (PEPFAR)-supported States in Nigeria. Data on patient background, HIV and TB care, and TB treatment outcomes were collected using an Excel abstraction template. The data was analyzed using SPSS and an association was examined using a chi-square test while binary logistic regression was used to determine predictors of TB treatment outcomes (P< 0.05).

**Results:**

two thousand six hundred and fifty-two co-infected patients participated in the study. The mean age of participants was 37 ± 14 years. A majority had TB treatment success (cured = 1059 (39.9%), completed = 1186 (44.7%)). Participants who had pulmonary TB, virally suppressed and commenced isoniazid (INH) before TB diagnosis were more likely to have a favorable TB treatment outcome compared to those who had extrapulmonary TB (AOR = 7.110, 95% CI = 1.506 - 33.565), virally unsuppressed (AOR = 1.677, 95% CI = 1.036 - 2.716) or did not commence INH before TB diagnosis (AOR = 1.486, 95% CI = 1.047 - 2.109).

**Conclusion:**

site of infection, immune status, exposure to ART, and INH prophylaxis were found to predict TB treatment outcomes among PLHIVs. Stakeholders should ensure early commencement of ART and INH prophylaxis for PLHIVs.

## Introduction

Tuberculosis (TB) and Human Immunodeficiency Virus (HIV) remain major public health threats throughout the world and worse in middle and low-income countries, especially when they co-exist in susceptible individuals despite the availability of proven effective treatments [1-3]. HIV infection increases a person´s susceptibility to TB disease by 20 to 30 times [4], which explains why the HIV epidemic is contributing significantly to the increasing prevalence of TB in many nations in the sub-Saharan African region [5]. Despite government and donor interventions in TB control all over the world, TB still ranks as one of the top 10 single causes of death globally, above HIV [2]. In 2019, TB disease affected about 10 million people globally, with 25% of the disease coming from Africa, second only to South-East Asia (44%) [2]. Nigeria is still the sixth-highest TB burden country, accounting for about 1 in 22 TB cases [2]. This is unarguably a great burden considering its high population and fragile healthcare system [6].

The strong synergistic association between tuberculosis (TB) and HIV is well-reported in the literature as a major challenge to patient survival. This has been described as a “catastrophic comradeship” [7]. Globally, the proportion of HIV-positive TB patients who died during treatment was 11%, similar to previous years and about three times the level among all new and relapse cases (4%) [1,2]. According to the 2018 TB global report, only nine of the 30 high TB burden countries reached or exceeded a 90% treatment success rate set a target for TB treatment outcome [1]. TB/HIV co-infection is largely responsible for the poor treatment success rate among TB patients due to the development of drug-resistant TB or Immune Reconstitution Inflammatory Syndrome (IRIS) [7].

Nigeria, with an estimated population of about 200 million people, is one of the countries in sub-Saharan Africa that bears the highest burden of HIV epidemics in the world and the current HIV prevalence in the general population is 1.4% [8]. Also, according to the World Health Organization (WHO) report, Nigeria has the highest burden of TB in Africa and ranks sixth among the 30 countries of the world with the highest TB burden [1], while the disease is the major driver of HIV epidemics. An estimated 63,000 Nigerians living with HIV/AIDS develop TB, while about 39,000 die from the disease, each year [2]. Tuberculosis (TB) remains a curable infectious disease if patients with drug-susceptible TB are treated on time with sustained and uninterrupted therapy for a specified duration [9]. Despite this, TB worsened by the HIV pandemic has proven difficult to eliminate, especially in developing countries [1]. The integration of TB and HIV/AIDS programmes activities in some countries have yielded good results [10]. However, in practice, many developing countries are yet to have effective and well-coordinated TB/HIV programmes that can reduce the morbidity and mortality associated with the diseases. Unfortunately, the two infectious diseases with a dual-directional relationship still constitute public health burdens to resource-limited nations [11]. The treatment of TB infection accounts for an important control measure, especially in patients co-infected with HIV [12].

Evaluating the treatment outcome of tuberculosis and identifying the associated factors should be an integral part of tuberculosis treatment. In several high TB burden countries, the quality of TB treatment outcome reporting has been low, creating gaps in national TB data and making it difficult to understand the factors associated with treatment outcomes [13]. Some of the factors that have been found to influence treatment outcome from an institution-based cross-sectional study include the place of residence, baseline weight, and experience of side effects [14], while there have been changes in drug options for the treatment of HIV which could be another factor that could affect TB treatment outcomes [15]. A facility-based study conducted on factors affecting TB treatment in Anambra and Oyo States found that many facilities in Anambra recorded poor treatment outcomes compared with that of Oyo State while the geographical location of the respondents was found to be associated with TB treatment outcomes [16]. Another study conducted in Ogun State among TB/HIV co-infected patients elicited facility type and being newly registered for TB treatment for the first time were predictors of TB treatment outcomes [17].

A deeper understanding of factors that are associated with and predicted TB treatment outcomes among People Living with HIV (PLHIV) using TB/HIV service delivery data from selected high volume Antiretroviral Therapy (ART) sites will enhance the development of strategies to improve TB treatment outcomes among PLHIVs. It will also provide a platform to step up the commitments and actions needed for the global TB epidemic control, especially among PLHIVs [18]. This study, therefore, examined the various TB treatment outcomes and factors that predicted outcomes of TB treatment among PLHIVs in the study location.

What are the common TB treatment outcomes among PLHIVs in the study locations? What are the TB treatment success rates among the same population? What factors are associated with the TB treatment outcomes? These questions will be answered from the findings of this study. Being a multicenter study, this work would provide a rich and diverse data source for a robust analysis and rich findings. This multicenter study will provide a diversified participant pool and enhanced external validity even though the study only involves PLHIVs.

## Methods

**Study design:** this study employed a cross-sectional design. A review of programme data of TB-HIV co-infected patients who had TB treatment across all high-volume comprehensive Antiretroviral Therapy (ART) sites with 500 patients or more in seven States being supported by the US Center for Disease Control and Prevention (CDC) through funds from the US President's Emergency Plan for AIDS Relief (PEPFAR) was carried out.

**Study setting:** Nigeria is located in West Africa. Nigeria is bordered by the Niger Republic, the Atlantic Ocean, the Benin Republic, the Republic of Cameroon, and Chard in the North, South, West, and East respectively. The country has six geopolitical zones which are: North East, North Central, North West, South West, South East, and South-South. The study was carried out in Oyo, Ogun, Ondo, Ekiti, Osun, Plateau, and Benue States. While Oyo, Ogun, Osun, Ekiti, and Ondo are located in Southwest Nigeria, Plateau, and Benue are located in the north-central part of the country. The service delivery data of PLHIVs who had TB/HIV coinfection between 1^st^ October 2017 and 30^th^ September 2021 were included in the study. The high-volume comprehensive ART sites selected also have a Directly Observed Therapy (DOT) clinic as a one-stop-shop (OSS) which affords TB/HIV, coinfected patients, to access dual health services in a clinic visit. HIV prevalence is higher in the Northcentral at 2.0% compared to the Southwest, which has a 1.1% prevalence [8]. Based on the Nigeria HIV/AIDS Indicator and Impact Survey (NAIIS) conducted in 2018, Benue State has the highest HIV prevalence of 5.3%, while Plateau, Ogun, Oyo Osun, Ondo, and Ekiti have a prevalence of 1.6%, 1.4%, 0.9%, 0.9%, 1.1%, and 0.8% respectively [8].

**Study population/participants:** the study population was TB/HIV coinfected patients in high-volume facilities across seven CDC/PEPFAR-supported States. The study participants include patients who had TB treatment with documented outcomes from October 1^st^, 2017, to 30^th^ September 2021. However, TB/HIV coinfected patients still on treatment or without documented outcomes were excluded from the study. Benue, Plateau, Oyo, Osun, Ondo Ogun, and Ekiti States have 10, 13, 8, 5, 4, 8, and 2 high-volume sites respectively. The number of PLHIVs accessing ART services across the States varies: as at the end of September 2021, Benue and Plateau had 223, 481 and 47,742 patients respectively while Oyo, Ogun, Osun, Ondo, and Ekiti provide comprehensive HIV services for 27,211; 24,365; 11,662; 14,067 and 5,578 respectively.

The comprehensive TB/HIV services provided in selected sites include HIV testing services, TB screening of all PLHIVs, Anti-retroviral treatment and Isoniazid Preventive Therapy (IPT) provision, and directly observed therapy services for PLHIV that are co-infected with tuberculosis. All the comprehensive sites maintain a comprehensive electronic database, alongside regular paper-based files where patients' clinical information is recorded on every clinic visit.

**Definitions of outcome variables:** cured: a pulmonary TB patient with bacteriologically confirmed TB at the beginning of treatment who was smear- or culture-negative in the last month of treatment and on at least one previous occasion. Treatment completed: a TB patient who completed treatment without evidence of failure but with no record to show that sputum smear or culture results in the last month of treatment and on at least one previous occasion were negative, either because tests were not done or because results are unavailable. Treatment failure: a TB patient whose sputum smear or culture is positive at month five [5] or later during treatment. Lost to follow-up: a TB patient who did not start treatment or whose treatment was interrupted for two [2] consecutive months or more. Defaulter: a patient who had been on treatment for at least four [4] weeks and whose treatment was interrupted for eight or more consecutive weeks [8]. Died: a TB patient who dies for any reason before or during treatment. Not evaluated: a TB patient for whom no treatment outcome is assigned. This includes cases transferred out to another treatment unit, as well as cases for whom the treatment outcome is unknown to the reporting unit. Treatment success: the sum of cured and treatment completed.

**Bias:** the study selected only patients with definitive TB treatment outcomes and whose data were uploaded on the Electronic Medical Record (EMR) at the time of data abstraction. The study also did not abstract data of TB/HIV co-infected patients with Multidrug-Resistant TB (MDR-TB) because MDR-TB treatment facilities are usually domiciled outside ART data was not accessible at the time of data abstraction.

**Samples size:** all TB/HIV coinfected patients that commenced TB treatment with documented TB treatment outcomes in EMR and other HIV/TB service delivery registers from 1^st^ October 2017 to 30^th^ September 2021 we sampled across all tier 1 ART sites.

**Data abstraction and management:** data were collected using an Excel abstraction template that elicited information on socio-demographics, HIV care history, TB care history, and TB treatment outcome of TB-HIV co-infected patients between 1^st^ October 2017 and 30^th^ September 2021 from the EMR. Treatment outcome was dichotomized into favorable treatment outcome (TB treatment completed and TB cured) and unfavorable (dead, lost to follow-up, and failed treatment). End-to-end encryption was done during data transmission from the health facilities to the programme staff and the data analyst to ensure the privacy and confidentiality of patient information. Furthermore, each study participant was assigned a unique identifier, and personal identifiers were removed from the dataset.

**Data analysis and statistical method:** independent variables measured include age, sex, type TB diagnostic evaluation, patient ART experience, ART regimen, based line CD4 count, current viral load suppression status, and INH prophylaxis. The dependent variables were TB treatment outcomes which were, completed, cured, died, loss to follow-up, and treatment failure. Data were cleaned and analyzed using SPSS IBM version 20 (IBM Corp, Armonk, NY, USA) and presented using frequency and 2 by 2 contingency tables. Chi-square analysis was done to examine the association between respondent´s treatment outcomes and study co-variates such as respondent´s sociodemographic characteristics, HIV care history, and TB care history. Binary logistic regression was also used to determine the adjusted odds ratio (AOR) and 95% confidence interval (CI) to identify the predictors of TB treatment outcomes. The level of significance for each test was set at P<0.05.

**Ethical consideration:** ethical approval for the study was sought and obtained from the Ethical Review Committee of the Department of Research, Planning, and Statistics of the Oyo State Ministry of Health for ease of administration (ref no: AD 13/479/4434^A^). Furthermore, the study utilized secondary data from a project approved by the Human Subject Unit of the US Center for Disease Control and Prevention (CDC) with non-research program monitoring protocol with the approved number of the Center for Global Health (CGH) Project tracking ID 2019-197 and also approved by APIN Public Health Initiatives Institutional Research Board (APIN IRB) for implementation across APIN supported States. Also, end-to-end encryption was done during data transmission from the health facilities to the programme staff and the data analyst to ensure the privacy and confidentiality of patient information. Furthermore, each study participant was assigned a unique identifier, and personal identifiers were removed from the dataset.

## Results

**Background characteristics (demographic and clinical history):** a total of 2,652 TB-HIV co-infected patients were involved in the study. The mean age of the study participants was 37 ± 14 years. About half of them were females (52.9%) who were previously on antiretroviral treatment (59.0%). A Larger percentage were on dolutegravir-based therapy (53.8%) and HIV is currently virally suppressed (89.1%) (Table 1).

**Table 1:** respondent’s background characteristics and clinical history

Variable (s)	Frequency (n = 2652)	Percent
**Age group**		
0-19	176	6.6
20-39	1203	45.4
40-59	1101	41.5
60 and above	172	6.5
**Sex**		
Male	1248	47.1
Female	1404	52.9
**Diagnostic evaluation**		
GeneXpert	1998	75.3
Chest X-ray	486	18.3
Microscopy	105	4.0
Sputum AFB	23	0.9
*Others	40	1.5
**ART experience**		
New ART clients	1087	41.0
Prior ART clients	1565	59.0
**ART regimen**		
DTG-based therapy	1426	53.8
Efavirenz based therapy	975	36.8
Nevirapine based therapy	141	5.3
**Others	110	4.1
**Regimen started first (ART or TB)**		
TB drug	2095	79.0
HIV drug	557	21.0
**Baseline CD4 cell count (n = 1420)**		
Less than 200 cells/ml	635	44.7
200-500 cells/ml	539	38.0
Greater than 500 cells/ml	246	17.3
**Current viral load suppression status (n = 2120)**		
Suppressed	1888	89.1
Unsuppressed	232	10.9
**Was INH commenced before TB diagnosis? (n = 2029)**		
No	820	40.4
Yes	1209	59.6

* others include biopsy and clinical diagnosis; **others include lopinavir/ritonavir (LPV/r) and atazanavir/ritonavir (ATV/r)-based therapies; AFB: acid-fast bacteria; ART: antiretroviral therapy; DTG: dolutegravir; TB: tuberculosis; INH: isoniazid

**Participants´ tuberculosis treatment outcome:** the majority of participants had favorable TB treatment outcomes (cured = 1059 (39.9%), completed = 1186 (44.7%)), while 293(11.0%), 93(3.5%) and 21(0.8%) were reported dead, loss to follow-up and failed treatment respectively (Table 2).

**Table 2:** respondent's TB treatment outcome

Variable (s)	Frequency (n = 2652)	Percent
Completed	1186	44.7
Cured	1059	39.9
Died	293	11.0
Loss to follow-up	93	3.5
Failed	21	0.8

TB: tuberculosis

**Factors associated with tuberculosis treatment outcome among study participants:** a higher percentage of participants who had pulmonary TB (84.9%, p < 0.01), on dolutegravir-based ART (86.6%; p = 0.03), with CD4 T-cell count greater than 500 cells/ml (88.2%; p = 0.05) had favorable TB outcome compared to their counterpart who had extrapulmonary TB, on other ART regimen or with CD4 cell count of 500 and below respectively. Similarly, participants who were HIV virally suppressed (88.6%, p < 0.01) and who had INH commencement before TB diagnosis (88.0%, p < 0.01) had favorable TB treatment outcomes compared to those who were virally unsuppressed or who did not commence INH before TB diagnosis (Table 3).

**Table 3:** association between TB treatment outcome and study independent variables

Variable (s)	TB treatment outcome		Total
	Completed/cured (n = 2245)	Died/failed/LTFU (n = 407)	
	Frequency (%)	Frequency (%)	
**Age group (in completed years)**			
0-19	145 (82.4)	31 (17.6)	176
20-39	1036 (86.1)	167 (13.9)	1203
40-59	925 (84.0)	176 (16.0)	1101
60 and above	139 (80.8)	33 (19.2)	172
**Sex**			
Male	1053 (84.4)	195 (15.6)	1248
Female	1192 (84.9)	212 (15.1)	1404
**TB site**			
Pulmonary	2207 (84.9)	392 (15.1)	2599
Extrapulmonary	38 (71.7)	15 (28.3)	53
**ART experience**			
New ART clients	914 (84.1)	173 (15.9)	1087
Prior ART clients	1331 (85.0)	234 (15.0)	1565
**ART regimen**			
DTG-based therapy	1235 (86.6)	191 (13.4)	1426
Efavirenz based therapy	803 (82.4)	172 (17.6)	975
Nevirapine based therapy	116 (82.3)	25 (17.7)	141
Others	91 (82.7)	19 (17.3)	110
**Regimen started first**			
TB drug	1769 (84.4)	326 (15.6)	2095
HIV drug	476 (85.5)	81 (14.5)	557
**CD4 cell count (n = 1188, 232)**			
Less than 200 cells/ml	517 (81.4)	118 (18.6)	635
200-500 cells/ml	454 (84.2)	85 (15.8)	539
Greater than 500 cells/ml	217 (88.2)	29 (11.8)	246
**VL suppression status (n = 1857, 263)**			
Suppressed	1672 (88.6)	216 (11.4)	1888
Unsuppressed	185 (79.7)	47 (20.3)	232
**History of INH commencement before TB diagnosis (n = 1727, 302)**			
No	663 (80.9)	157 (19.1)	820
Yes	1064 (88.0)	145 (12.0)	1209

* significant at p-value < 0.05; TB: tuberculosis; LTFU: loss to follow-up; ART: antiretroviral therapy; DTG: dolutegravir; VL: viral load; INH: isoniazid

**Predictors of favorable tuberculosis treatment outcome:** study participants who had pulmonary TB, and who had INH commencement before TB diagnosis had a higher likelihood of having favorable TB treatment outcomes compared to those who had extrapulmonary TB (adjusted odd ratio (AOR) = 6.79, 95% CI = 1.39 - 33.07), or those who did not have INH commencement before TB diagnosis (AOR = 1.50, 95% CI = 1.06 - 2.14). Furthermore, those who were virally unsuppressed were likely to have favorable TB treatment outcomes compared to those virally suppressed (AOR = 0.59, 95% CI = 0.37 - 0.96) (Table 4).

**Table 4:** examine predictors of TB treatment outcome among study respondents

Variable (s)	Adjusted OR	95% CI for AOR	
		Lower	Upper
**Age group**			
0-19	1		
20-39	1.51	0.77	2.98
40-59	1.06	0.55	2.05
60 and above	0.90	0.39	2.05
Sex (female vs male)	1.07	0.75	1.52
TB Site (pulmonary vs extra-pulmonary)	6.79	1.39	33.07
**CD4 cell count**			
< 200 cells/ml	1		
200 - 500 cells/ml	1.35	0.92	1.99
> 500 cells/ml	1.66	0.99	2.77
Viral load suppression status (unsuppressed vs suppressed)	0.59	0.37	0.96
History of INH commencement before TB diagnosis (yes vs no)	1.50	1.06	2.14
**ART regimen**			
DTG-based therapy	1		
EFV-based therapy	0.71	0.48	1.05
NVP-based therapy	0.96	0.39	2.35
Others	0.82	0.33	2.00

* significant at p-value < 0.05; TB: tuberculosis; INH: isoniazid; ART: antiretroviral therapy; DTG: dolutegravir; EFV: efavirenz; NVP: nevirapine

## Discussion

This study assessed the various outcomes of TB treatment and associated factors among people with HIV/TB co-infections in APIN/CDC/PEPFAR-supported sites in Nigeria. The treatment success rate (TSR) for this study was 84%. This value is close to what was reported by a study done in Abeokuta, Ogun State (83.5%) [17] and another study conducted in Oyo State (82%) [16]. The TSR in this study was higher than what was reported by studies done in Lagos (78%) [18] and Anambra State (57.5%) [16]. Our finding of 84% TSR among TB/HIV co-infected patients is only slightly below the recommended target of 85% by the National Tuberculosis and Leprosy Control Programme [19] and the World Health Organization (WHO) [20]. However, according to the 2020 WHO Global Tuberculosis Report, the global treatment success rate for HIV-associated TB cases among the 2018 patient cohort was 76% [2]. Additionally, the TSR in this study was higher than reported studies from Cameroon (78.6%) [21], Ethiopia (58%) [22] and Western Ethiopia (60.7%) [23], Malaysia (53.4%) [24], Brazil (55%) [25] and India (66%) [26]. The high treatment success rate may be attributable to the integration of TB and HIV services in these centres and the availability of DOTS services while another reason might be due to close monitoring by the implementing partners and the states´ TB control programmes.

Although our study did not find age and sex to be significantly associated with TB treatment outcomes, however, site of TB infection, ART regimen, patients CD4 count, and viral load suppression status were found to be associated with outcomes of TB treatment in PLHIVs. This is in contrast to the findings reported in a study done in Lagos, Nigeria, that age >20 years decreased the likelihood of successful treatment [18]. However, another study done in Ethiopia found the contrary, that age was an associated factor for successful TB treatment outcomes, where the age group of 25-49 years was associated with favorable outcomes [22]. Similarly, a study done in Cameroon found that being female was significantly associated with a successful TB treatment outcome [21], while an Ethiopian study also found that female patients were more likely to have a successful treatment outcome compared with male patients counterparts [22].

In this study, patients with good immunologic and virologic profiles had better TB treatment outcomes than those with poor immunologic and virologic profiles. Our study found that the higher the CD4 count, the more likely the treatment success; patients with a CD4 count: <200 cells/mm^3^ had 81% TSR, those with a CD4 count of 200-500 cell/mm^3^ had TSR of 84% and those with CD4 count of > 500 cells/mm^3^ had 88% TSR. In the same light, among those with documented and valid viral load results, patients with viral suppression (viral load <1000 copies/ml) had 88.6% TSR while unsuppressed patients had 79.7%. Studies have found that the immunosuppression experienced by TB patients as a result of low CD4 count and advanced HIV disease at the time of HIV diagnosis increases the risk of mortality and poor treatment outcomes [18,27,28]. This agrees with our findings that the more severe the immunosuppression, the more the likelihood of poor treatment outcomes.

In addition, study participants who had pulmonary TB had a higher likelihood of having favorable TB outcomes compared to those who had extrapulmonary TB. Eighty-four percent of those with pulmonary TB had treatment completion or cure as compared to the 71.7% in PLHIVs with extrapulmonary TB. This is not surprising as extrapulmonary TB may be associated with more immunosuppression compared to pulmonary TB [14,29]. The same was found in a study conducted in North Central Nigeria [30]. However, this is in contrast with the findings of a study done at the Bowen University Teaching Hospital, Ogbomoso, Nigeria, where they found extrapulmonary TB associated with good treatment outcomes [31].

It is of note that patients' exposure to Isoniazid Preventive Therapy (IPT) and ART before TB infection was found to be a statistically significant predictor of the favorable TB treatment outcome. INH has been documented in many pieces of literature to prevent TB among PLHIV [32-34]. WHO recommends that PLHIV who screen negative for any of the symptoms of current cough, fever, weight loss, and night sweats are unlikely to have TB and therefore should receive at least 6 months of IPT as part of a comprehensive package of HIV care [35,36]. However, some people have argued that continuous use of INH as prophylaxis may increase INH-resistant TB strains in undiagnosed active TB or inadequate treatment [37]. This study, however, found that prior INH prophylaxis exposure before TB infection was associated with good TB treatment outcomes, while a study concluded that INH resistance concerns are insufficient to rule out the use of IPT when there are active case findings and effective TB treatment [33].

**Study limitation:** this retrospective study relied on secondary data. The data was limited to whatever was already documented in the patient folders and registers at TB and ART clinics in the selected sites. While these are validated data, it is important to note there may be other factors that associated with the variables especially because this was a multicenter study. For example, the viral suppression rates varied from site to site due to the varying of effectiveness of adherence support. This may even affect the treatment completion rates. Furthermore, variables such as behavioral, social, and economic factors, which might affect treatment outcomes directly or indirectly, were not routinely documented in the patients´ records. This is the major limitation of this study.

## Conclusion

In this study, there was a high TB treatment success rate among TB-HIV co-infected patients who were placed on TB treatment. In addition, high CD4 count (greater than 500 cells/mm^3^) at the commencement of ART, DTG-based regimen, exposure to INH prophylaxis prior to TB diagnosis, and viral suppression were all found to be associated with better TB treatment outcomes. Therefore, early diagnosis of HIV, before CD4 falls below 200 cells/mm^3^ as in advanced HIV disease, would enhance the likelihood of good treatment outcomes in PLHIVs who may be diagnosed with TB later. Similarly, there was a higher likelihood of TB Treatment success among PLHIVs with pulmonary TB who had INH prophylaxis prior to TB diagnosis. Therefore, clinicians should ensure TB and INH (tuberculosis preventive therapy) screening for every PLHIV at every clinic visit for TB prophylaxis, early diagnosis, and prompt treatment.

### What is known about this topic

- *Tuberculosis remains the most common opportunistic infection and a major cause of death among people living with HIV;*
- *Tuberculosis (TB) treatment outcomes among the general population;*
- *Isoniazid prophylaxis prevents TB among PLHIV*.

### What this study adds

- *The majority of TB/HIV co-infected patients with Pulmonary TB and prior INH prophylaxis exposure had TB treatment success;*
- *Immune status, drug regimen, TB prophylaxis, viral suppression status, and site of TB infection were associated with TB treatment outcomes among PLHIVs*.
